# Enhancing sustainability in self-compacting concrete by optimizing blended supplementary cementitious materials

**DOI:** 10.1038/s41598-024-62499-w

**Published:** 2024-05-29

**Authors:** Abdul Aziz, Syed Saqib Mehboob, Aisha Tayyab, Diyar Khan, Khizar Hayyat, Afsar Ali, Qadir Bux Imran Latif Qureshi

**Affiliations:** 1 Department of Civil Engineering, University of Engineering and Technology, Taxila, Pakistan; 2 Department of Industrial Engineering, University of Engineering and Technology, Taxila, Pakistan; 3 Doctoral School, Silesian University of Technology, Akademicka 2a, 44-100 Gliwice, Poland; 4 Department of Civil and Environmental Engineering, College of Engineering and Architecture, University of Nizwa, Birkat-al-Mouz, Nizwa, 616 Oman

**Keywords:** Self-compacting concrete (SCC), Metakaolin, Lime powder, Response surface method (RSM), Optimization, ANOVA, Engineering, Civil engineering

## Abstract

Within concrete engineering, the uptake of self-compacting concrete (SCC) represents a notable trend, delivering improved workability and placement efficiency. However, challenges persist, notably in achieving optimal performance while mitigating environmental impacts, particularly in cement consumption. However, simply reducing the cement content in the mix design can directly compromise the structural-concrete requirements. Towards these challenges, global trends emphasize the utilization of appropriate waste materials in blended concrete. This study explored a promising strategy by integrating supplementary cementitious materials (SCMs) to contribute to the United Nations' Sustainable Development Goals (SDGs) in addition to the engineering contributions. It suggests an optimal combination of Metakaolin (MK) and Limestone Powder (LP) to partially substitute cement. The research methodology employs the response surface method (RSM) to systematically explore the ideal ingredient ratios. Through a comprehensive analysis of orthogonal array of 16 mixes, encompassing both mixture and process variables, this study aims to explain the effects of MK and LP addition on the rheological and mechanical properties of SCC with varying cement replacement levels. In terms of mixture constituents, the total composition of cement, MK, and LP was fixed at 100%, while coarse aggregate (CA), fine aggregate (FA), and the water-to-binder ratio were held as process variables. In order to assess the rheological properties of the mix-design, various tests including slump flow, L-box, and sieve segregation were conducted. Additionally, to evaluate mechanical strength, samples were tested for compressive strength at both 7 and 28 days. Findings from the experiments reveal higher concentrations of MK result in reduced workability and hardened properties. Through RSM-based designed experimentation covering both rheological and mechanical aspects, it is observed that the optimal cement replacement level lies between 40 and 55%. The findings of this study contribute to the advancement of sustainable and structurally robust concrete practices, offering insights into the optimal utilization of SCMs to meet both engineering requirements and environmental sustainability goals.

## Introduction

In the twenty-first century, according to the United Nations annual report, waste-generation has potentially increased leading to environmental issues^1^. In conjunction, in the construction sector the excessive utilization of sand and gravel during production of concrete has not only significantly decreased natural resources but also CO_2_ emissions has raised serious concerns regarding global warming^2^. From the last two decades, construction sector is considered as crucial domain, and the constructional projects cannot be overlooked. Cement is a vital part of infrastructural construction activities and produces significant economic impact. According to reports worldwide approximately production of cement is three billion tons every year^3^. Therefore, the cement industry is facing exceptional challenges due to production of Ordinary Portland Cement (OPC) related to climatic changes as well as ailing economy of world in recent times. The inflation rate in prices of cement to alarming level during past few years has raised the need to explore other natural cost-effective materials for the substitution of cement^4^. To overcome these barriers, researchers are recommending utilization of eco-friendly supplementary cementitious materials (SCMs) either partially- or fully-replacing OPC in concrete construction^5^. The global campaigns are aiming to boost production and utilization of environment-friendly substantial construction material that can substitute conventional concrete materials^6^. The Sustainable Development Goals (SDGs) set by the United Nations aim to implement the 2030 Agenda worldwide, striving for a more sustainable and equitable future for all inhabitants of Earth. Core principles of this eco-friendly initiative include resource conservation, pollution reduction, energy efficiency, waste minimization, and the preservation of the planet's ecological equilibrium^7,8^. Conventional concrete faces challenges such as (i) difficulty in placing concrete in densely reinforced sections and (ii) contributing to the depletion of natural resources. An eco-friendly and potential alternative to OPC concrete is Self-Compacting Concrete (SCC) made from waste products of other industries. In this aspect SCC has gained enormous popularity^9,10^. Self-compacting concrete (SCC) plays a pivotal role in civil engineering structures by offering numerous advantages. Its ability to flow into confined spaces and around reinforcement simplifies the construction process, reducing labor-intensive tasks associated with traditional concrete placement. Moreover, SCC provides a smoother finish, enhancing the aesthetics of various civil structures and minimizing the need for additional surface treatments. The homogeneous and well-compacted nature of SCC contributes to improved durability by reducing the risk of voids and imperfections that could compromise long-term performance. Additionally, SCC’s uniform distribution of aggregates and de-creased likelihood of segregation enhance structural integrity, crucial for the longevity and stability of diverse civil engineering projects^11^. The designing of SCC, optimal design mix is a challenging task as a wide variety of factors affect the properties of SCC^12^. Therefore, to replace conventional concrete it is needed to explore all possible existing alternative raw materials that can serve as sustainable alternative replacement to OPC^13^.

The utilization of supplementary cementitious materials (SCMs) offers a promising opportunity for reducing cement usage in concrete production. These materials encompass a wide range, including Fly Ash Class F (FA-F), Ground Granulated Blast Furnace Slag (GGBS), Silica Fume (SF), Rice Husk Ash (RHA), Marble Powder, among others, offer several advantages due to their unique chemical composition and pozzolanic reactivity. Few studies^14,15^ have been performed on utilizing other industrial wastes like Waste Foundry Sand (WFS) and Marble Powder (MP) waste in the development of SCC. The use of aluminosilicate SCMs enhances the performance of concrete by improving early-age strength development, reducing permeability, and enhancing durability against various deteriorative mechanisms, including sulfate attack and alkali-silica reaction (ASR). Moreover, their fine particle size distribution facilitates better packing and densification of the concrete matrix, leading to improved mechanical properties and overall performance.

By incorporating these SCMs into concrete mixes, a portion of the cementitious binder can be replaced, resulting in reduced cement consumption, and associated environmental benefits, such as lower CO_2_ emissions. In the realm of such materials the one of two key points that was considered in this study was to target a SCM which exhibits high aluminosilicate content. However, despite possible advantages, there may exist challenges related to optimizing the incorporation of highly aluminosilicate SCMs in concrete mixes, including issues related to workability, setting time, and long-term performance. Addressing these challenges through comprehensive research and RSM-based experimental design for the mix ratio is essential to fully realize the potential benefits of these materials in sustainable concrete construction.

Moreover, its optimal replacement with Ordinary Portland Cement (OPC) is also a matter to investigate for its implication. Table 1 summaries the chemical compositions of several SCMs from various published studies and compared with the composition of the Ordinary Portland Cement (OPC), Metakaolin (MK) and Lime powder (LP) used in this study as a contribution towards the optimal and sustainable mix design of SCC.

**Table 1:** Chemical compositions of SCMs.

References	Material	Chemical composition (%)										
		SiO_2_	Al_2_O_3_	Fe_2_O_3_	MgO	CaO	SO_3_	K_2_O	Na_2_O	TiO_2_	Cl	LOI
This Study	OPC	22	5.5	3.5	1.7	63.47	1.82	1.00	0.20	–	–	0.64
	LP	8.2	1.53	0.96	1.27	71.39	–	–	–	–	–	–
	MK	45.1	39.4	0.57	–	1.6	–	–	0.8	–	–	13.94
^16^	FA-F	58.55	28.2	3.44	0.32	2.23	0.07	1.26	0.58	–	–	4.17
^17^	MP	0.7	0.29	0.12	0.23	55.49	–	1.8	2.44	–	–	42.83
^18^	GGBS	40.1	6.0	2.0	4.7	42.2	0.15	1.2	–	1.2	–	–
^19^	SF	92.26	0.89	1.97	0.96	0.49	0.33	1.31	0.42	–	–	–
^20^	RHA	94	1.2	0.37	0.6	2.93	0.3	0.5	–	–	–	–
^17^	BP	54.62	9.6	4.14	4.66	12.8	0.66	1.62	0.84	–	–	9.94

The selection of Metakaolin (MK), rich in aluminosilicate content, is based on its potential to yield satisfactory results in self-compacting concrete (SCC). Table 1 illustrates the chemical composition of Metakaolin, revealing a significant presence of over 84% silica and alumina content. The ratio of SiO_2_/Al_2_O_3_ is measured at 1.14. MK is chosen for its ability to impart desirable characteristics of structural strength, essential for typical field applications, while also offering favorable workability properties. Despite the rationale behind this choice of supplementary cementitious materials (SCMs), due to the highly sensitive nature of SCC, even slight variations in constituent materials can lead to significant changes in concrete properties, potentially compromising structural concrete requirements. This underscores the importance of precise mix design methodologies to achieve optimal performance. One such method gaining prominence is the response surface method (RSM), which systematically explores the effects of various factors on concrete properties, allowing for the identification of the most favorable mix compositions. It is a mathematical and statistical techniques useful for the modelling and analysis of problems in which a response of interest is influenced by several variables^21^. It is powerful technique for optimization of chemical reactions and engineering processes to evaluate the effect of SCMs in SCC development^22,23^. Several studies^24–27^ have highlighted the efficacy of RSM in optimizing SCC formulations using different SCMs.

This research was motivated by the goal of advancing green construction practices and enhancing the practicality of concrete usage in the field. Utilizing Metakaolin (MK) in self-compacting concrete (SCC) to achieve optimal strength became the focus, aiming to provide engineers and researchers with a reliable database. Likewise, in their research^28^, the authors have demonstrated commendable progress in the development of self-compacting concrete (SCC) utilizing Metakaolin, employing a strength-based mixture design approach. The methodology involves experimental trials to optimize various constituents. Additionally, the research highlights that the maximum 28-day compressive strength, reaching approximately 89 MPa, was achieved at a Metakaolin replacement level of approximately 15%. But such traditional experimental approaches for assessing multiple parameters in SCC properties often involve extensive trial experiments. For instance, if there are four levels and five parameters, then 4^5^ = 1024 trial mix of experiments are needed to examine the impact of each parameter and each level^29^. This requires an enormous commitment of time and money. Therefore, a second key point is setting a suitable strategy for experiment design to explore the impact of these parameters with fewer experiments. Hence, the Response Surface Method (RSM) was employed in this study as an optimization technique to overcome these challenges. The objective was to develop a cost-effective and time-efficient eco-friendly design mix for SCC. By replacing cement with both Metakaolin and Lime Powder simultaneously, the study aimed to achieve an optimized SCC with desirable characteristics. Furthermore, it is postulated that the incorporation of these supplementary cementitious materials could contribute to sustainability by mitigating the environmental impact associated with cement production.

## Data source and research methodology

**Figure 1:**
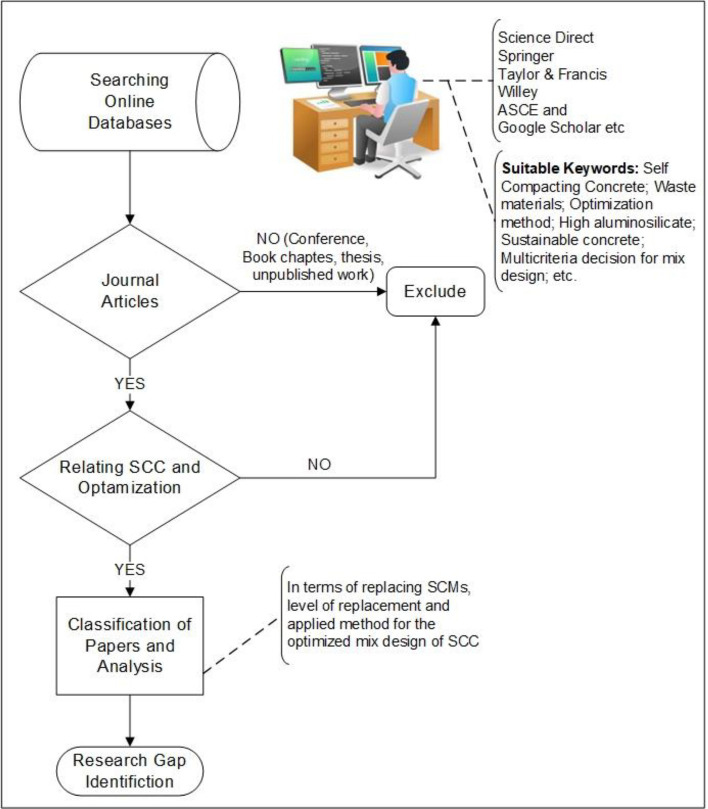
Flow chart of research methodology.

Previously published research articles on self-compacting concrete (SCC) have significantly influenced the utilization of various supplementary cementitious materials (SCMs), and alternative raw material substitutes. These studies have distinctly explored the developmental stages of SCC, alongside the requisite constituent materials. These investigations have encompassed evaluations of both the plastic and hardened state properties of SCC. Moreover, there is a pressing need to explore into the utilization of high replacement levels (i.e., above 40%) of Metakaolin and limestone powder blends in SCC development, employing Response Surface Method (RSM) optimization techniques, and conducting comparison of different SCMs of varying aluminosilicate percentages. The primary objective of this methodology, as depicted in Fig. 1, is to dissect SCMs rich in aluminosilicate content, crucial for manufacturing SCC.

## Materials and methods

Metakaolin (Shaheen Mining Corporation); Cement (Fuji Cement Company Ltd); Limestone powder (Naeem Brothers Marble, Granite and Chakwal Stone Works); Coarse aggregate (Khanpur); Fine aggregate (Lawrencpur); Superplasticizer (Sika Pakistan). Software-Design Expert (DX^®^), Ver 22 (Stat-Ease, Inc., Minneapolis, USA) was employed to analyse the data for optimization.

### Materials

#### Metakaolin

Metakaolin is obtained of Kaolin when burnt at 500–900 °C properties shown in Table 2^30^. The pozzolanic property of metakaolin is reported to be greater than silica fume^31,32^ and capable of enhancing the cementitious property of aggregates^33,34^. The metakaolin observed to improve the concrete mechanical properties^35,36^ that minimized the adverse effects produced by traditional mineral admixture (ordinary coal fly ash and blast furnace slag) to the concrete^35^. Metakaolin is reported to increases strengths of all basic properties viz. compressive strengths, flexure strengths, split strengths, tensile strengths. It increases resistance to chemical attack and reduces alkali silica reactivity. It enhances workability and finishing of concrete. It reduces shrinkage due to particle packing. It can be used in formation of high performance, high strength, and lightweight concrete, precast and poured-mold concrete^37,38^.

**Table 2:** Metakaolin properties.

Physical properties	Results
Specific gravity	2.40 to 2.60
Color	Off white, Gray to buff
Physical form	Powder
Average plastic size	< 2.5 μm
Brightness	80–82 hunter L
BET	15 m/g
Specific surface	8–15 m/g

##### Cement

Cement is an essential and significant binding material. Cement is a widely used binding material in the whole world during construction. In this experimental procedure cement named "Fauji" which is OPC, meets the requirement of ASTM C150 was used having properties mentioned in Table 3.

**Table 3:** Cement properties.

Physical properties	Results
Cement type	OPC
Normal consistency %	26.75
VICAT initial settling time (min)	99
VICAT final settling time (min)	227
Specific gravity	3.03
Le-Chatelier expansion (mm)	0
Fineness (by sieve)	92.75%

##### Lime powder

Lime Powder (LP) was produced by quarrying process of grinding and crushing of Lime Stone that is a non-pozzolanic material^39^. The LSP mineral composition exists in variety of forms such as calcite, aragonite, amorphous calcium carbonate and vaterite etc.^40^. The properties shown in Table 3. LSP exhibits filler effect due to which it is used in production of cement. LSP is reported to cause health hazards as well as pollution in environment^41^. Different stages of hydration, including dissolution, acceleration, dynamic balancing, and cemented stage, have been encountered in cement paste with a high LSP content^39^. It increases hydration, which results in strong early strength^42^. LSP is a low-cost, extremely effective concrete that lowers carbon dioxide emissions^43^ without compromising the strength characteristics^44^ and improves the sulphate resistance in CC^45^.

Concrete made from waste wood fiber and waste limestone powder is lightweight. When the amount of limestone powder in cement paste increases, carbo-aluminate is formed, increasing the need for CaCO_3_. LSP is more widely used in concrete due to its inexpensive cost and increased availability. Table lists all of the chemical features of the binder^46,47^. All binder chemical properties have been mentioned in Table 4.

**Table 4:** Lime powder properties.

Physical property	Value
Color	Light grey–dark grey
PH (of water slurry)	11–13
Specific density g/cm	2.65–3.0
Bulk density (in loose state) g/cm	0.7–1.1
Chlorides content %	0.004–0.034

##### Coarse aggregate

**Figure 2:**
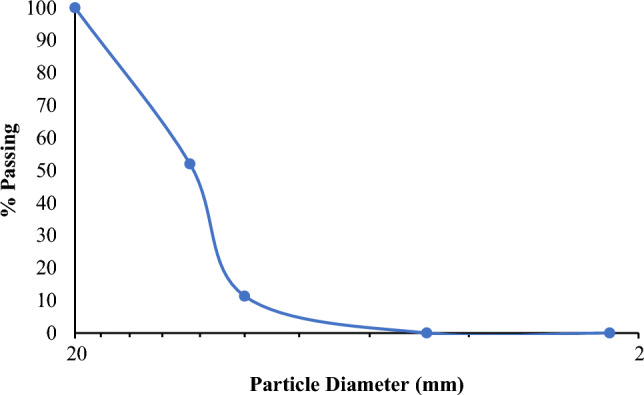
Coarse aggregate gradation curve.

Construction projects typically use coarse aggregate (CA) to add strength. Because it comes in a variety of sizes, aggregate is a versatile material. This is a portion of stones that has been crushed; it is typically mined. The investigation used aggregate that was smaller than 19 mm in size and complied with ASTM C 33M (Designation: C618-12a Standard Specification for Coal Fly Ash and Raw or Calcined Natural Pozzolan for Use in Concrete) having the following properties given in Tables 5, 6 and Fig. 2 display the CA grading curve.

**Table 5:** Properties of coarse aggregates.

Properties	Coarse aggregates
Surface texture	Rough
Particle shape	Angular
Specific gravity	2.66

**Table 6:** Coarse aggregate grading.

BS (mm)	ASTM (inch)	Mass retained (g)	Retained (%)	Cumulative passing (%)
20	3/4″	0	0	100
12.5	1/2″	1440	48	52
10	3/8″	1220	40.66	11.34
4.75	3/16″	340	11.34	0
2.25	Pan	0	0	0
	Total	3000

##### Fine aggregate

**Figure 3:**
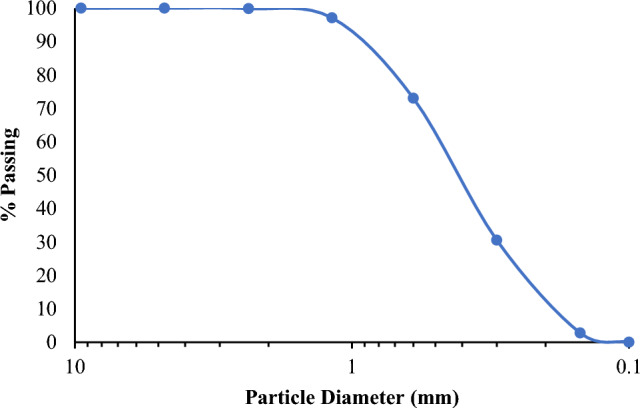
Fine aggregate gradation curve.

Sand, an ingredient of SCC mixes and a necessary component for concreting, is referred to as fine aggregate (FA). The substance used in the experiment was smaller than 4.75 mm in size. This substance, which is called a filler, is used just to fill the gaps left in the concrete matrix. In this study, FA that complies with ASTMC33 standards was employed and has some properties mentioned in Tables 7, 8 and Fig. 3 shows the FA gradation curve.

**Table 7:** Properties of fine aggregate.

Properties	Fine aggregates
Surface texture	Smooth
Particle shape	Rounded
Specific gravity	2.732

**Table 8:** Fine Aggregate Gradation.

BS (mm)	ASTM (inch)	Mass retained (g)	Retained (% )	Cumulative passing (%)
9.5	3/8″	0	0	100
4.75	#4	0	0	100
2.36	#8	1.2	0.12	99.88
1.18	#16	27.7	2.77	97.11
0.6	#30	240.6	24.06	73.05
0.3	#50	424.9	42.49	30.56
0.15	#100	277.8	27.78	2.78
0.1	PAN	27.8	2.78	0

##### Superplasticizer

Admixture Superplasticizer (SP) is a chemical used to enhance the strength of concrete and water reducing agent. Admixtures (SP) used during construction to attain desired results at minimum cost. During this research high water reducing SP named Viscocrete-3110 (“Sika^®^ ViscoCrete^®^-3110 W) was used to attain high workability or flowability. Physical properties are mentioned in Table 9.

**Table 9:** Superplasticizer physical properties.

Physical properties	Results
Color	Yellowish
Form	Liquid
Density (kg/m^3^)	1080
Specific weight (g/cm^3^)	1.17

### Concrete mix design and specimen preparation

For finalizing the confined number of mixes levels of parameters in Table 10, the 16 No of trail mixes in Table 11 were carefully selected. Based on the results of all the trail mixes the behavior of the concrete with addition of MK and LP is studied. Finally, 16 mixes were concluded for the further experimental study named as M1, M2, M3, M4, M5, M6, M7, M8, M9, M10, M11, M12, M13, M14, M15 and M16.

**Table 10:** Levels of parameters.

Level	Cement replacement %	W/B ratio	Coarse aggregates (kg/m^3^)	Binder content (kg/m^3^)	Fine aggregates (kg/m^3^)
1	40	0.32	769.86	450	690.00
2	45	0.38	756.68	475	678.18
3	50	0.45	743.56	500	666.37
4	55	0.5	730.31	525	654.55

**Table 11:** Mix design.

Mix. ID	Binder content (kg/m^3^)	Cement % replaced level	Cement (kg/m^3^)	MK (%)	MK (kg/m^3^)	LP (%)	LP (kg/m^3^)	W/B	Water (kg/m^3^)	CA (kg/m^3^)	FA (kg/m^3^)	SP (kg/m^3^)
M1	450	40	270	30	135	10	45	0.32	144	769.86	690	4.00
M2	475	40	285	25	118.75	15	71.25	0.38	180.5	756.68	678.18	4.10
M3	500	40	300	20	100	20	100	0.45	225	743.56	666.37	4.20
M4	525	40	315	15	78.75	25	131.25	0.5	262.5	730.31	654.55	4.30
M5	500	45	275	30	150	15	75	0.32	160	756.68	654.55	4.10
M6	525	45	288.75	25	131.25	20	105	0.38	199.5	743.56	690	4.20
M7	450	45	247.5	20	90	25	112.5	0.45	202.5	730.31	678.18	4.30
M8	475	45	261.25	35	166.25	10	47.5	0.5	237.5	769.86	666.37	4.00
M9	525	50	262.5	30	157.5	20	105	0.32	168	743.56	678.18	4.20
M10	450	50	225	25	112.5	25	112.5	0.38	171	730.31	666.37	4.30
M11	475	50	237.5	40	190	10	47.5	0.45	213.75	769.86	654.55	4.00
M12	500	50	250	35	175	15	75	0.5	250	756.68	690	4.10
M13	475	55	213.75	30	142.5	25	118.75	0.32	152	730.31	666.37	4.30
M14	500	55	225	45	225	10	50	0.38	190	769.86	654.55	4.00
M15	525	55	236.25	40	210	15	78.75	0.45	236.25	756.68	690	4.10
M16	450	55	202.5	35	157.5	20	90	0.5	225	743.56	678.18	4.20

*CA* coarse aggregate, *FA* fine aggregates, *SP* superplasticizer.

All batches were mixed for five minutes by using mix design proportions for all mixes needs to be prepared. The number of specimens of each mix are prepared in cubes for each mix are casted 3 for each age testing 7 and 28 days. Total no of samples cast was 96 + 6 = 102. The 96 samples were for 16 trial mixes and 06 samples were for validation study.

The fresh properties tests i.e., slump flows, L-box and segregation resistance test were performed on completion of mixing time of each mix. All tests for fresh state properties were conducted following the methods given by the SCC Committee of EFNARC (2005). Fresh properties of mixes tested in this experimental study are slump flow, L-box test, and segregation resistance. The hardened properties that is compressive e strength at 7 and 28 days were also studied for each mix by using laminated wooden molds in concrete laboratory. These molds were firstly cleaned and then properly oiled. After oiling, molds were placed on a clean surface and filled under their weight without compaction. After casting the top surfaces of samples were leveled. Then all specimens were stored at room temperature until demolding. The steel molds were removed after one day and all specimens were placed for moist curing as per ASTM C192. The compressive strength was examined at 7 and 28 days respectively for each mix.

### Optimization RSM

Design Expert (DX^®^), Ver 22 (Stat-Ease, Inc., Minneapolis, USA) was employed to analyze the data for optimization. The data used were given in Table 12. The factors were divided into two categories i.e., mixture and process variables. The cement, metakaolin and lime were included as mixture components constraints with the total of 100 percent. While the W/B, coarse aggregate and fine aggregate were considered as process variables. The data were entered in customized design with blank sheet adjusted to mixture components and process factors with each of 3 variables. The details of the mixture and process components have been elaborated in Tables 13, 14 and 15. A model for each response was auto selected where the significant terms were automatically selected if they qualify a p value at alpha 0.1. The terms were also included if they were hierarchal. Using the above strategy model reduction is not required. A predictable model usable to navigate the design space was assessed based on the goodness of fit statistical criteria as mentioned below: A higher model F-value indicate a significant model a lower chance for F-value value occurs due to noise, p < 0.05 indicate significant model terms. The difference between the Predicted R^2^ and Adjusted R^2^ should be < 0.2 which indicates their reasonable agreement. The Adeq Precision, a measure of the signal to noise ratio is desired to be > 4 for an adequate signal. An equation in terms of coded factors was used to predict a response for given levels of each factor. The process factors and mixture components with high levels are coded as + 1, 0 and − 1 respectively while those with low levels are coded as 0 and − 1 respectively. The coded equation also helps identify the relative impact of the factors by comparing the factor coefficients. The further suitability of the model was also assessed using graphical criteria such as normal residual vs predicted, Cook’s distance and box cox graphs. The later graph was also used for the data transformation. The desirability of the response was given according to Table 12.

**Table 12:** RSM optimization input and output parameters.

Mix. ID	Mixture component = total 100			Factors			Properties				
	Cement (%)	Metakaolin (%)	Lime powder (%)	W/B	Coarse agg. (kg/cum)	Fine agg. (kg/cum)	Slump flow (mm)	Passing ability	Segregation resistance (%)	Compressive strength at 7 days (Mpa)	Compressive strength at 28 days (Mpa)
M1	60	30	10	0.32	769.86	690	545	0.160	19.76	16.20	18.82767
M2	60	25	15	0.38	756.68	678.18	645	0.212	1.13	15.04	19.71915
M3	60	20	20	0.45	743.56	666.37	800	0.345	33.40	16.39	18.88581
M4	60	15	25	0.5	730.31	654.55	835	0.846	60.00	14.85	18.18813
M5	55	30	15	0.32	756.68	654.55	415	0.308	1.32	13.46	20.20365
M6	55	25	20	0.38	743.56	690	770	0.257	58.18	14.61	21.34707
M7	55	20	25	0.45	730.31	678.18	560	0.188	5.36	14.86	18.38193
M8	55	35	10	0.5	769.86	666.37	747	0.470	94.48	12.03	14.42841
M9	50	30	20	0.32	743.56	678.18	450	0.300	0.88	8.43	13.74042
M10	50	25	25	0.38	730.31	666.37	590	0.150	11.56	7.69	14.16678
M11	50	40	10	0.45	769.86	654.55	865	0.250	54.5	7.97	11.42451
M12	50	35	15	0.5	756.68	690	620	0.800	11.04	10.53	12.48072
M13	45	30	25	0.32	730.31	666.37	400	0.380	1.70	7.85	14.070849
M14	45	45	10	0.38	769.86	654.55	405	0.250	1.02	6.59	8.16867
M15	45	40	15	0.45	756.68	690	450	0.530	2.30	5.97	8.76945
M16	45	35	20	0.5	743.56	678.18	840	0.833	25.98	7.46	9.1086
						Desirability	750	0.85	10	Max	Max

**Table 13:** Factors for optimization.

Component		Name	Units	Type	Sub Type	Minimum	Maximum
Mixture	A	Cement	%	Mixture		45	60
	B	Metakaolin	%	Mixture		15	45
	C	Lime powder	%	Mixture		10	25
	Total						100.00
Process	D	W/B		Numeric	Continuous	0.3200	0.5000
	E	Coarse agg	kg/cum	Numeric	Continuous	730.31	769.86
	F	Fine agg	kg/cum	Numeric	Continuous	654.55	690.00

**Table 14:** Projected responses with factors for optimization.

Sr	Name	Units	Observations	Minimum	Maximum	Mean	Std. Dev	Ratio
R1	Slump flow	Mm	16.00	400	865	621.06	169.48	2.16
R2	Passing ability	–	16.00	0.15	0.846	0.39	0.24	5.64
R3	Segregation resistance	%	16.00	0.88	94.48	23.91	28.53	107.36
R4	Compressive strength at 7 days	MPa	16.00	5.97	16.39	11.25	3.79	2.75
R5	Compressive strength at 28 days	MPa	16.00	8.16	21.34	15.12	4.35	2.61

**Table 15:** Design constraints.

Low limit	Constraint			High limit
45.00	≤	A: Cement (%)	≤	60.00
15.00	≤	B: Metakaolin (%)	≤	45.00
10.00	≤	C: Lime Powder (%)	≤	25.00
		A + B + C	=	100.00

## Results and discussions

### Fresh properties results

#### Slump flow test

The workability of each mix was investigated by using slump cone test according to ASTM C143. Slump cone is placed over a 200 mm diameter circle on the base plate and filled with SCC mix in such a way that concrete is not leaked from the bottom of the cone without any vibration. The concrete remained in the cone for approximately 30 s, the cone is removed upwards without any interruption. When concrete touched the mark then measured the largest diameter in both directions by using measuring tape without disturbing base plate. The mean of the two largest diameters will be slump flow result and reported in millimeters. The slump flow of SCC with different proportions of cement and proposed contents of MK and LP are shown in Fig. 1. The incorporation of MK significantly reduced the workability due to its finest surface as due to finest surface of MK the required quantity of water increases to make it workable so that is why increase in quantity of MK causes reduction in workability. All mixes like M1, M5, M9 and M13 having w/b ratio of 0.32 have failed in slump flow values and mixes having higher replacement of cement i.e., 50% and 55% have been failed due to having higher content of Mk like M11, M14 and M15. The main reason for decreasing in slump flow value was MK particle size because the surface area of MK was more than the cement particles. Because it is spread out over a vast region and impedes the movement of fresh concrete a big surface area has a detrimental impact on the fresh and rheological qualities of concrete. Because of this slump, flow was reduced by raising MK in SCC.

AS per our site requirements the workability desired values have been taken 750 mm that is in between of minimum and maximum values so that during any conditions the design of SCC has flexibility of workability as during concrete work delay in mixing, placing and transporting has high margins to meet requirements making it more feasible in practical life.

#### L-Box test

The passing ability of SCC through L-Box test with different proportions of cement, MK and LP of all mixes has been checked by performing L Box test. First, the L-Box is placed in the level surface, then concrete is filled in L-Box and allowed to remain in this for approximately 60 s. After that, the gates are opened, and the concrete mix can pass through the vertical bars entered in the horizontal section. Blockage and segregation were recorded behind bars and in the horizontal section, if any. When the concrete stops moving in the horizontal section, then the concrete height is measured at three different locations in the horizontal section, and height is recorded as H2 in mm. The height of the concrete just behind the gate is recorded as H1 in mm. The blocking ratio is calculated as = H2/H1. In the experiment the passing ability values were found in EFNARC ranges of three mixtures M4, M12 and M16 having W/B ratio 0.5. It can be attributable to water cement ratio on higher side as compared to other mixes that made SCC more flowable due to its fluidity leading to decrease in blockage. The desired values having value of 0.85 have flexibility in ranges to make the concrete more flowable due to any delays in concrete pouring work making it practical in daily life.

##### Sieve segregation resistance test

To perform sieve segregation resistance test First of all, fill the sample container by (10 ± 0.5) Liters of concrete and fix the top cover of container. The sample is allowed to stand for 15 min without disturbance. Then place the sieve receiver on the weighing machine and its mass (Wp g) may be recorded. After that then place the sieve on receiver and again mass may be recorded accurately. After expiry of standup period remove top cover from container having sample and it may be noted that whether bleeding has occurred or not on surface of concrete. With the sieve accompanying receiver still placing on weighing machine, and with top of sample container (500 ± 50) mm above the sieve, pour immediately (4.8 ± 0.2) kg concrete (including any bleed water if appeared on concrete surface) onto the center of the sieve. Then the actual mass of concrete (WC g) on sieve may be recorded accurately. Actual mass value comes after deducting the mass of sieve along with receive from total mass of concrete that has been poured on the sieve center. The poured concrete may be allowed to stand for (120 ± 5) s and then remove sieve carefully in direction of vertically without any agitation. The mass of receiver and concrete that has passed into it from receiver may be recorded as (WPS g). The segregated portion (SR) may be calculated from following equation given below and report to the nearest 1%. SR = (Wps − Wp) 100/Wc %. Sieve segregation resistance values of mixes M3, M4 M6 M8, M11 and M16 were not in EFNARC limits due to having lesser viscosity as compared to other having dense viscosity shown Fig. 3. Both ages (7- and 28-days) compressive strength of mixes from M1 to M8 were found to be higher as compared to mixes M9 to M16 due to higher replacement of cement percentage. The desired value of segregation resistance of mid value of EFNARC values make it more feasible in applications having maximum tolerance in execution.

### Hardened properties results

#### Compressive strength test

To evaluate compressive strength three (150 × 150 × 150 mm) Cubes of each mix are casted and leave at the place of casting for hardening purposes (Raju and v Raghava Rao Me 2018). Casted samples leave for 24 h after demolding samples are placed in water for curing purposes. After curing, samples are tested in the compressive testing machine at 7 and 28 days. This chapter summarized all guidelines of SCC by Japan and EFNARC and all basic requirements of SCC mix. Tests of SCC in fresh state and hard state are explained in detail by following guidelines by EFNARC and ASTM. The compressive strength of SCC with different proportions of all mixes have been checked for 7 and 28 days respectively. The maximum compressive strength was to be of M6. The increase in strength can be attributed due to the addition of mineral admixtures i.e., MK pozzolanic and micro-filling features. It can be justified by the pore size reduction and refinement of crystal structure by addition of fine particles up to an optimized limit. It can be noted that with an increase ratio of MK the strength decreases by increasing cement replacement level.

### Optimization RSM method

#### Slump flow

The ANOVA (see Table 16) shows the model and the significant terms. The data was modeled by using the modified reduced Linear × 2FI. No data transformation was needed for this model. The Predicted R^2^ of 0.5801 was in a reasonable agreement with the Adjusted R^2^ of 0.7709 as the difference between above was < 0.2. The Adeq Precision ratio was 8.072 indicated an adequate signal, as it was > 4. This model can be used to navigate the design space. The F-value of 8.21 reflected the significance of the model. There is only a 0.40% chance that an above F-value could occur due to noise. p-values < 0.05 indicated only D was significant, while A, B were not significant, yet they were involved in the significant interactions, such as AD, BE, BF and DF (Table 16). The model terms bearing p > 0.10 were not significant yet included to support hierarchy. The final equation in Terms of L-Pseudo Components and Coded Factors was Slump flow (mm) = + 981.62A + 864.01B + 277.30D − 55.18E − 303.37AD − 444.51BE − 191.80BF − 161.29DF.

**Table 16:** ANOVA for significance of model.

Response	Source	Sum of squares	df	Mean square	F-value	p-value
Slump flow (mm)	Model (Reduced Linear × 2FI)	3.782E + 05	7	54,030.76	8.21	0.0040*
	^(1)^Linear Mixture	− 2.079E + 05	1	− 2.079E+05	− 31.59	1.0000
	D-W/B	1.427E+05	1	1.427E+05	21.69	0.0016*
	E-coarse agg	2485.20	1	2485.20	0.3777	0.5559^♣^
	AD	13,550.61	1	13,550.61	2.06	0.1892^♣^
	BE	40,616.67	1	40,616.67	6.17	0.0379*
	BF	64,131.63	1	64,131.63	9.75	0.0142*
	DF	80,537.49	1	80,537.49	12.24	0.0081*
Pass ability	Model (Quadratic × Quadratic model)	0.8345	7	0.1192	46.57	< 0.0001*
	^(1)^Linear Mixture	0.0483	2	0.0242	9.44	0.0079
	D-W/B	0.0246	1	0.0246	9.62	0.0146*
	AB	0.0955	1	0.0955	37.29	0.0003*
	BD	0.0622	1	0.0622	24.31	0.0011*
	D^2^	0.3405	1	0.3405	133.03	< 0.0001
	F^2^	0.0250	1	0.0250	9.75	0.0142
Segregation resistance	Model (Quadratic × 2FI model)	5.79	5	1.16	5.24	0.0128*
	^(1)^Linear Mixture	0.9523	2	0.4761	2.15	0.1669
	D-W/B	3.68	1	3.68	16.63	0.0022*
	AB	0.8292	1	0.8292	3.75	0.0816^♣^
Compression strength at 7 day	DF	1.38	1	1.38	6.23	0.0316*
	Model (Linear × linear)	198.93	3	66.31	48.08	< 0.0001*
	^(1)^Linear Mixture	193.80	2	96.90	70.26	< 0.0001
	CF	5.13	1	5.13	3.72	0.0778^♣^
Compression strength at 28 day	Model (Linear × linear)	250.29	3	83.43	30.09	< 0.0001*
	^(1)^Linear Mixture	226.05	2	113.02	40.77	< 0.0001
	D-W/B	24.24	1	24.24	8.74	0.0120*

*Significant mode or terms. ^♣^Non-significant terms, yet added to support hierarchy of the model.

**Figure 4:**
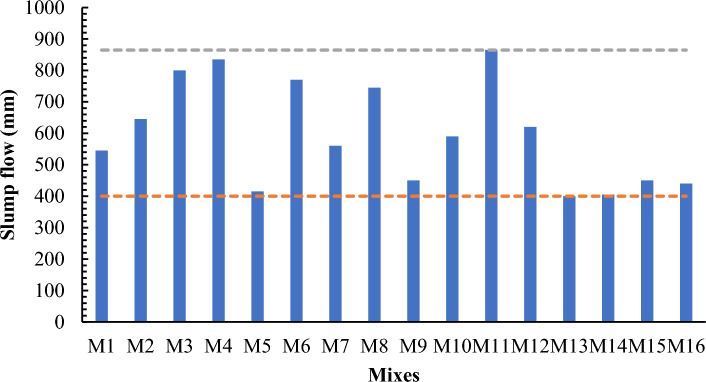
Slump flow results.

**Figure 5:**
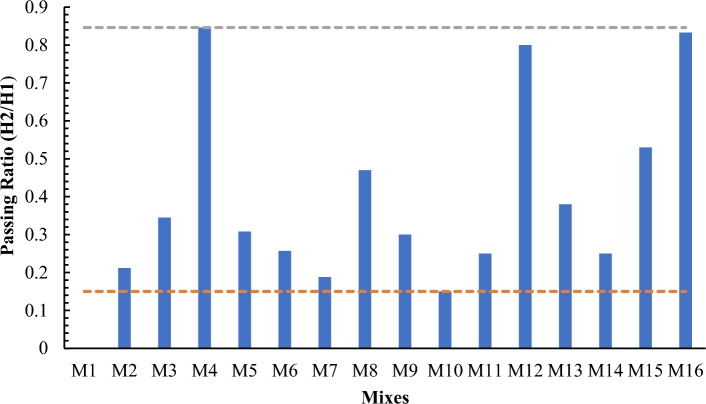
L-Box test results.

**Figure 6:**
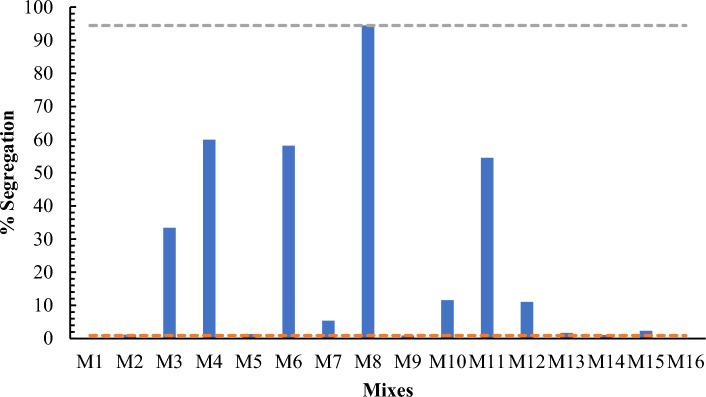
Sieve segregation resistance test of all mixes.

**Figure 7:**
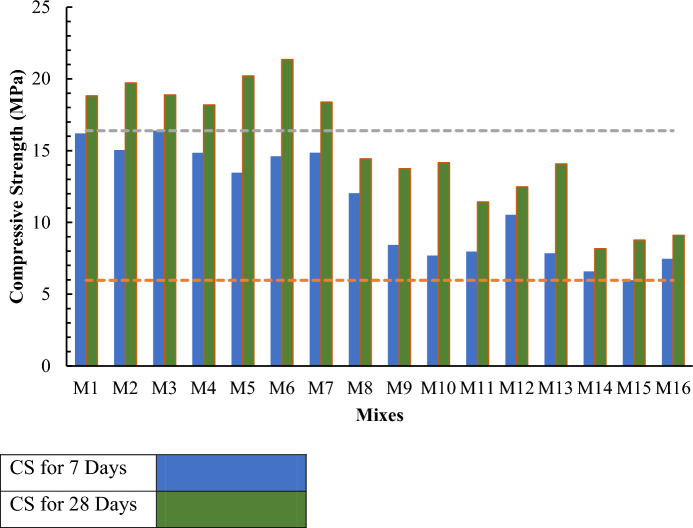
Compressive strength test results.

**Figure 8:**
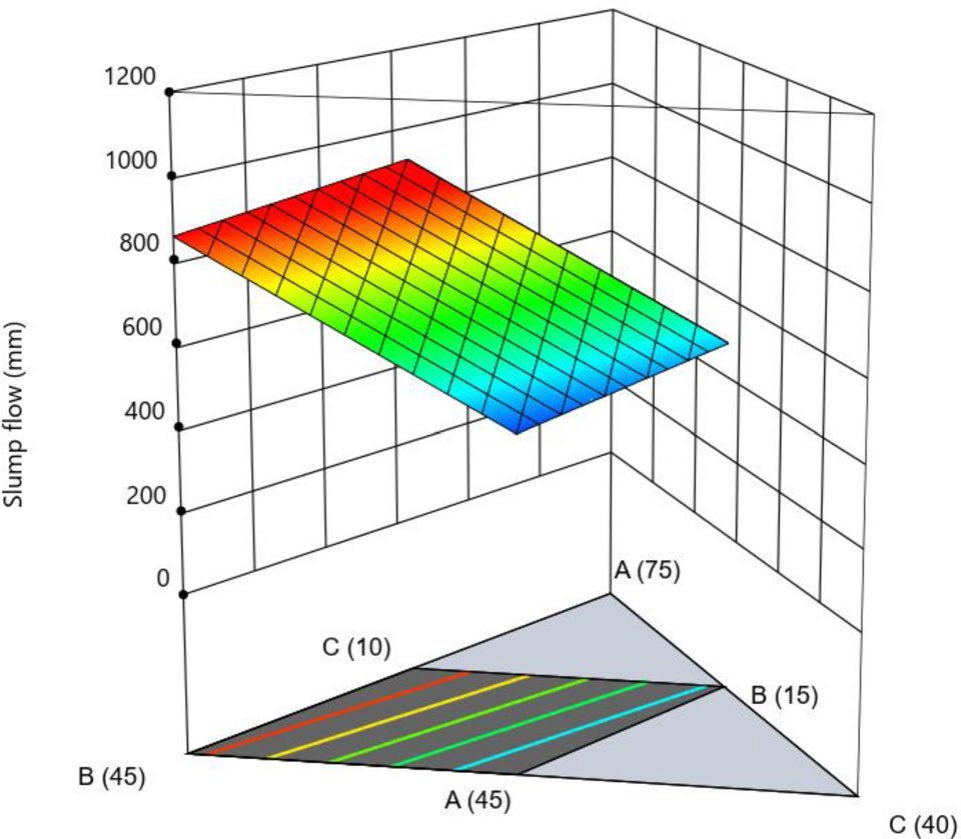
Combine effect of (**a**) cement, (**b**) metakaolin, (**c**) lime powder on slump flow.

**Figure 9:**
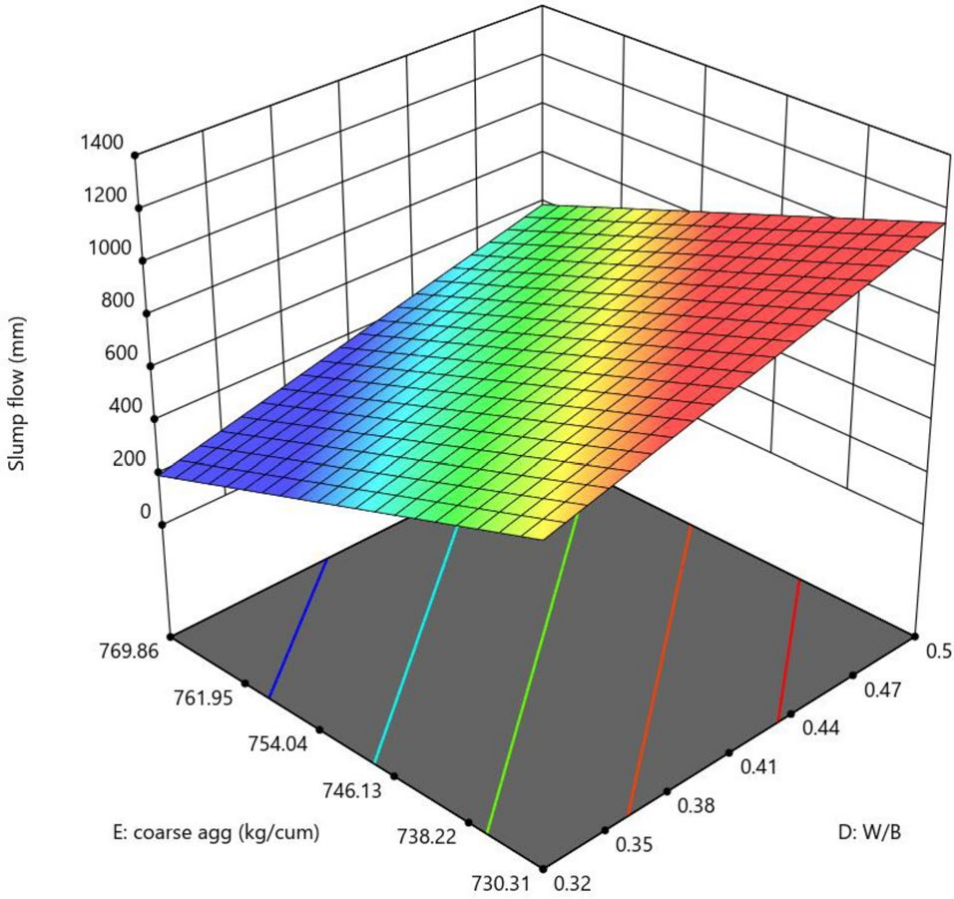
Combine effect of coarse aggregate and W/B ratio on slump flow.

**Figure 10:**
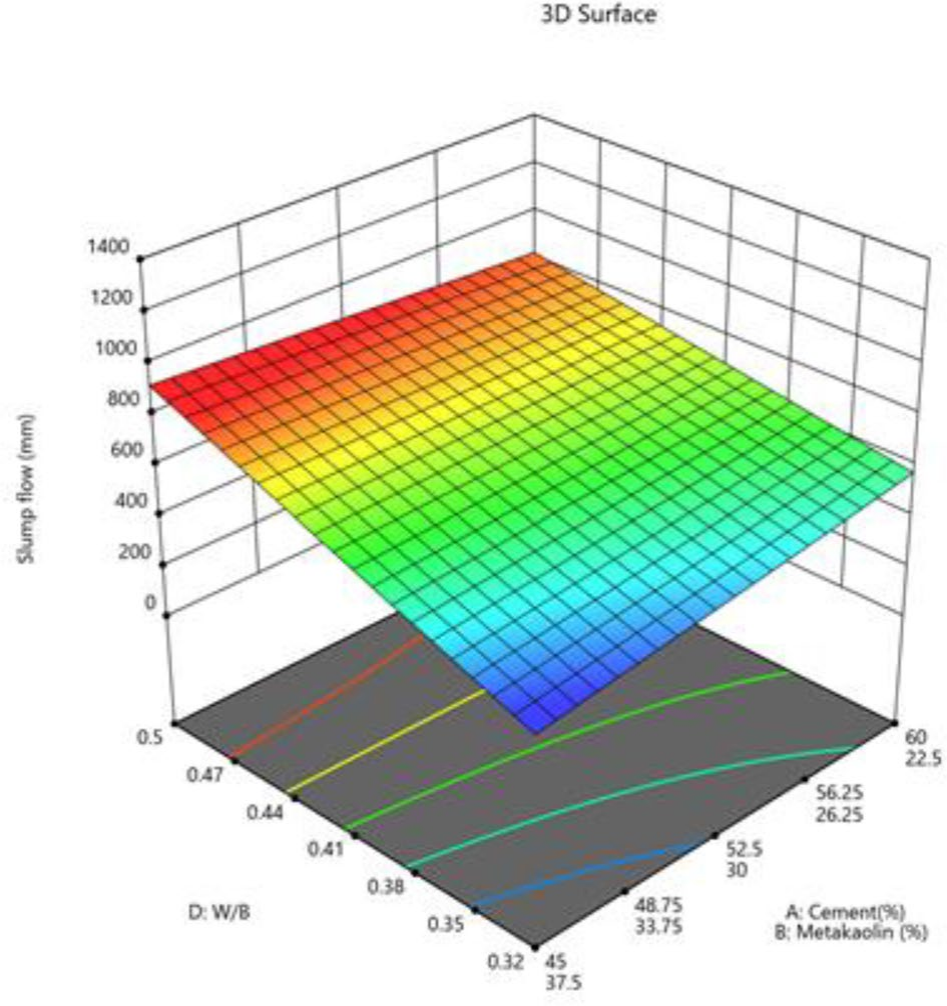
Combine effect of W/B ratio, cement, metakaolin on slump flow.

**Figure 11:**
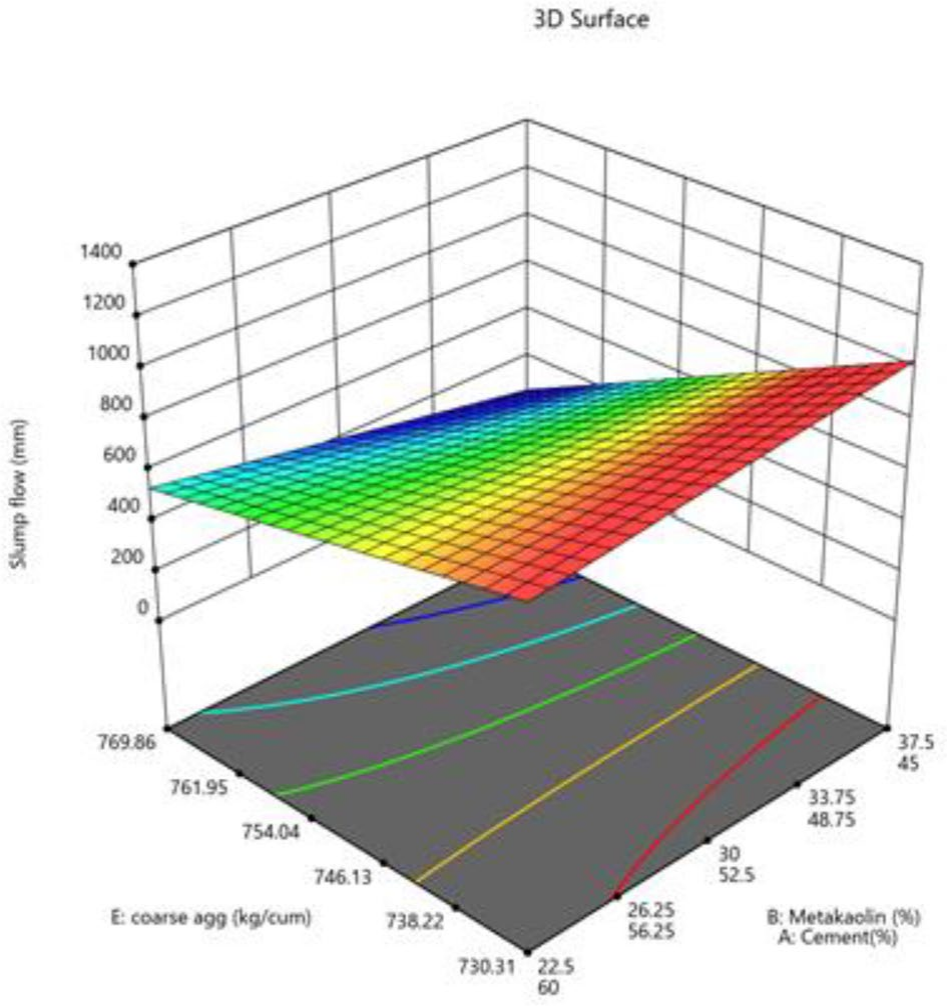
Combine effect of coarse aggregate, cement, metakolin on slump flow.

**Figure 12:**
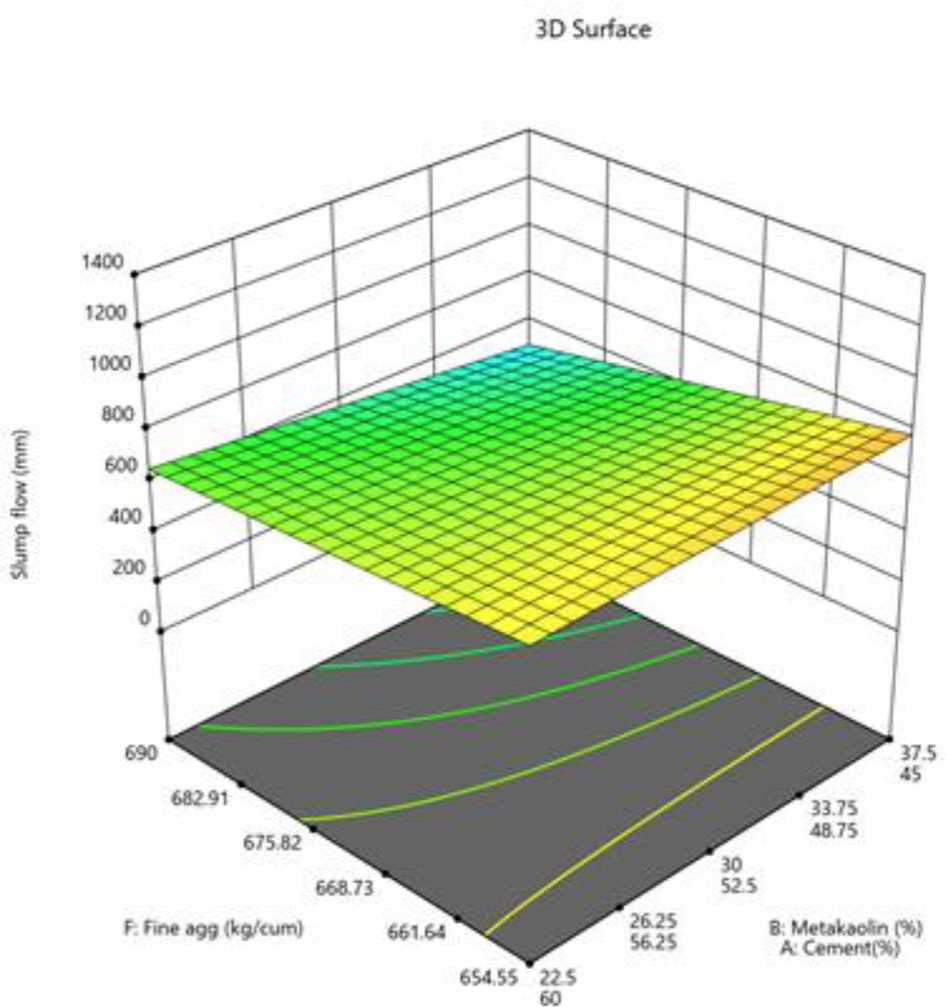
Combine effect of fine aggregate, cement, metakolin on slump flow.

**Figure 13:**
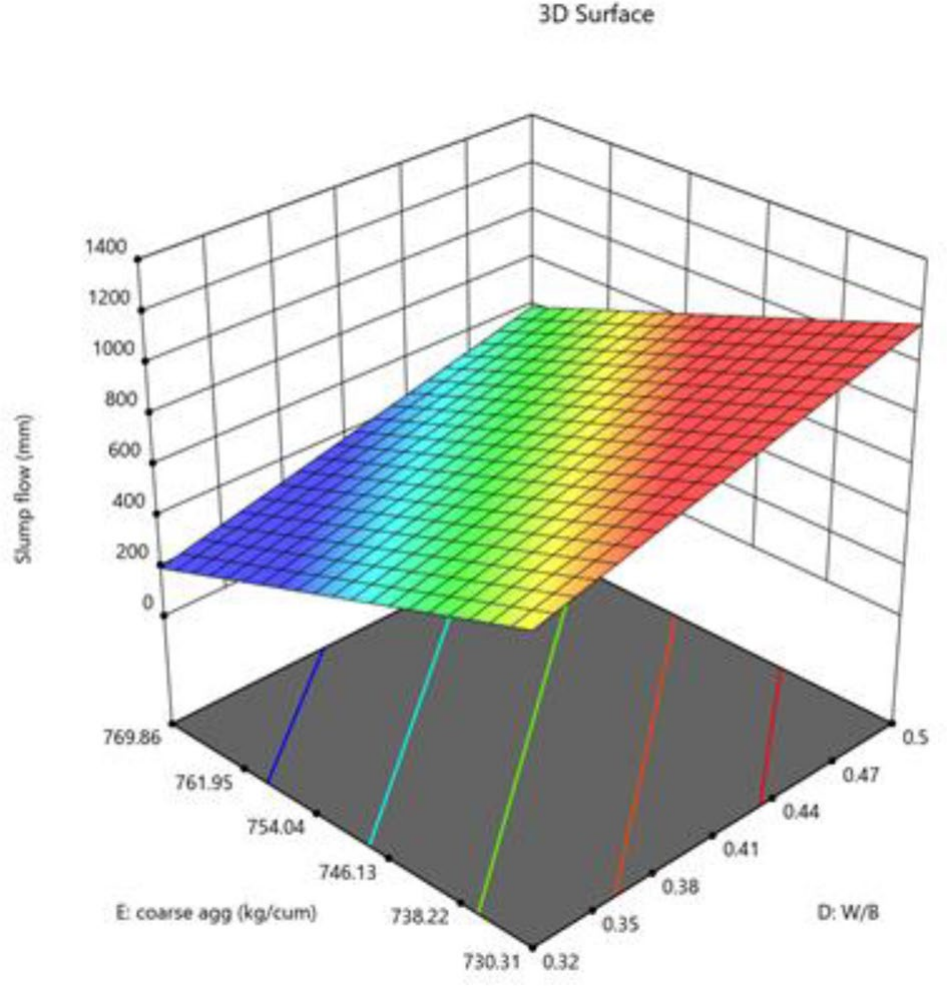
Combine effect of coarse aggregate, W/B ratio on slump flow.

The mixture components, such as cement, metakaolin and lime all have positive effects (as indicated by + symbol) on slump flow, yet most prominent effect was of cement (864), followed by metakaolin (277) and lime have no effect, as it has excluded from equation. Factor D (W/B ratio) E (coarse aggregate) and factor interactions, such as AD, BE, BF and DF affected negatively on slump flow. The response surface plots indicate combined effects of two factors on response Figs. 3, 4, 5, 6, 7, 8, 9, 10, 11, 12, 13.

#### Passing ability

**Figure 14:**
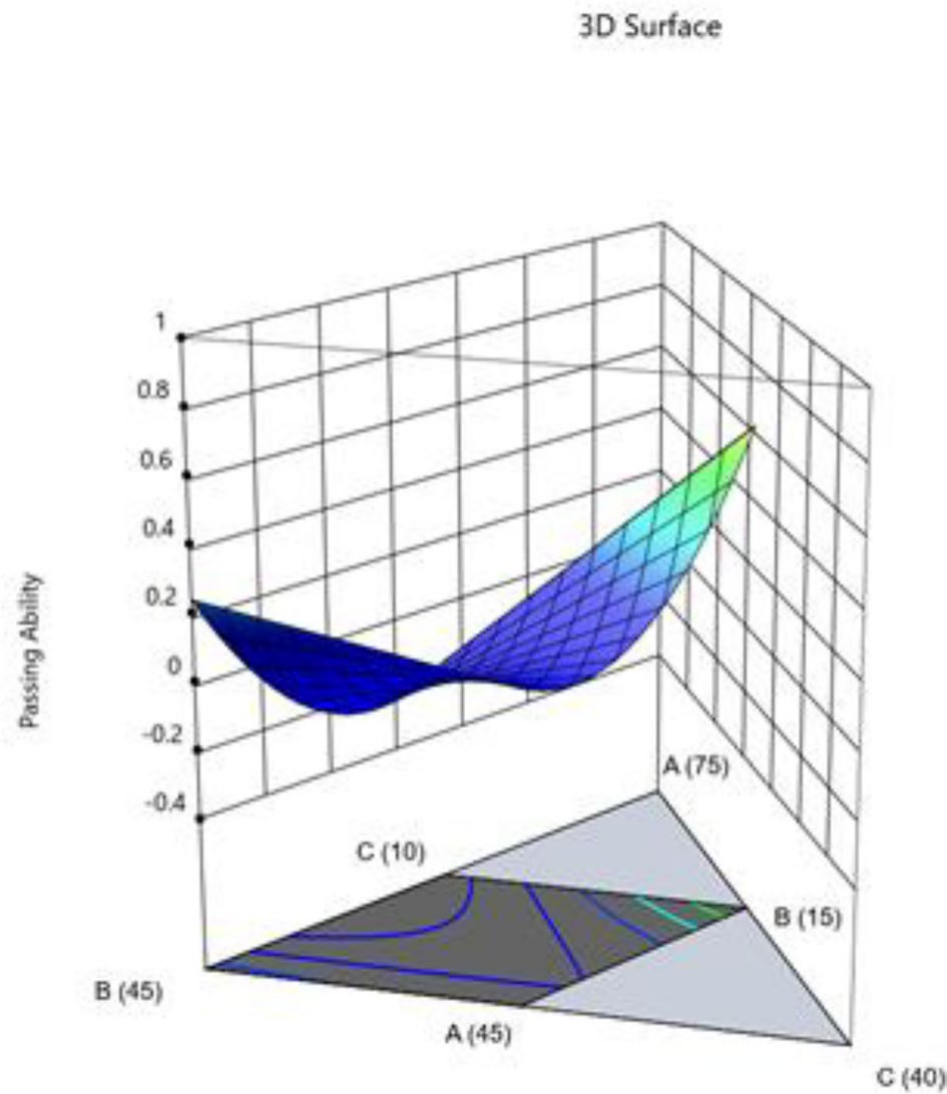
Combine effect of (**A**) cement, (**B**) metakaolin, (**C**) lime powder on passing ability.

**Figure 15:**
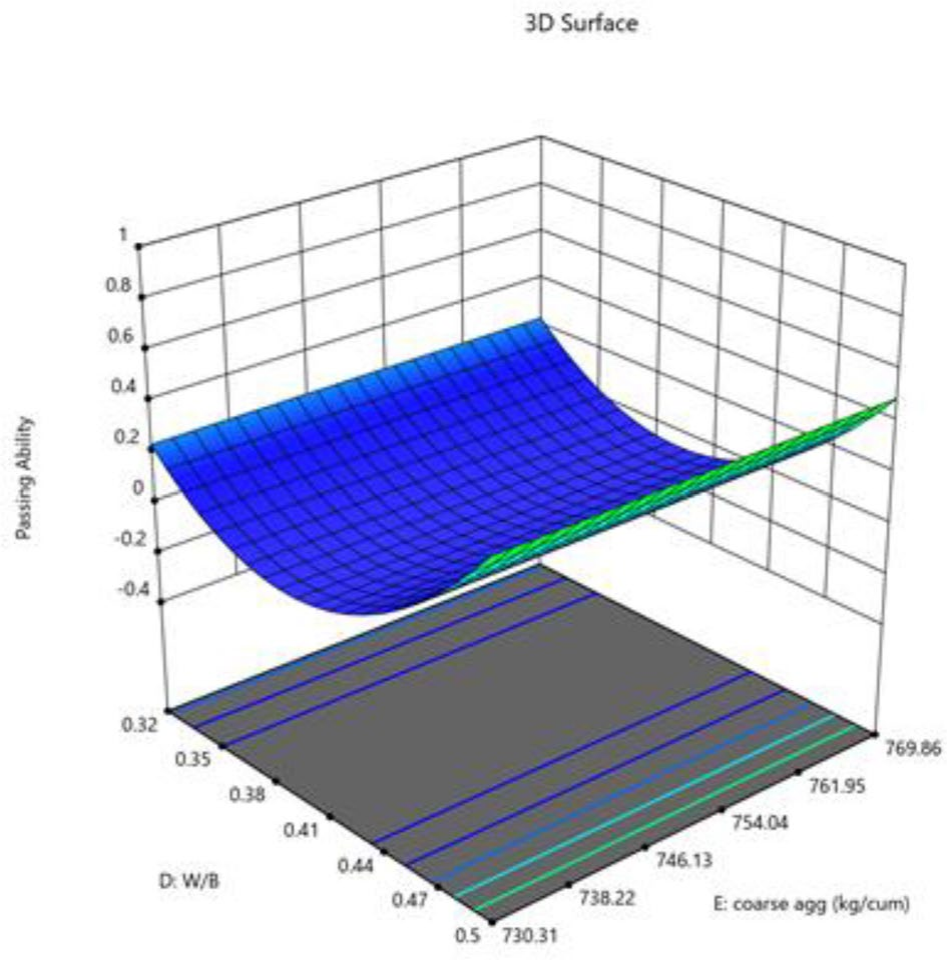
Combine effect of coarse aggregate, W/B ratio on passing ability.

**Figure 16:**
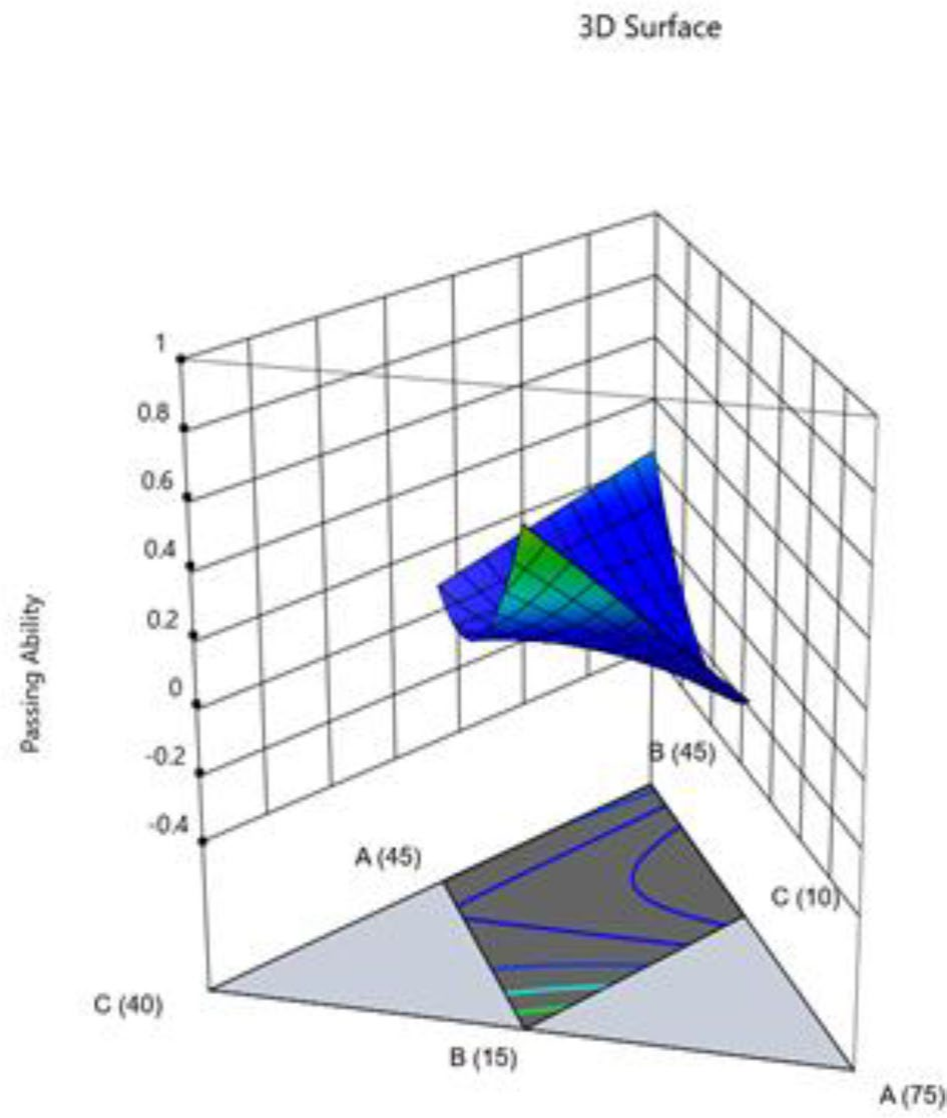
Combine effect of (**A**) cement, (**B**) metakaolin, (**C**) lime powder on passing ability.

**Figure 17:**
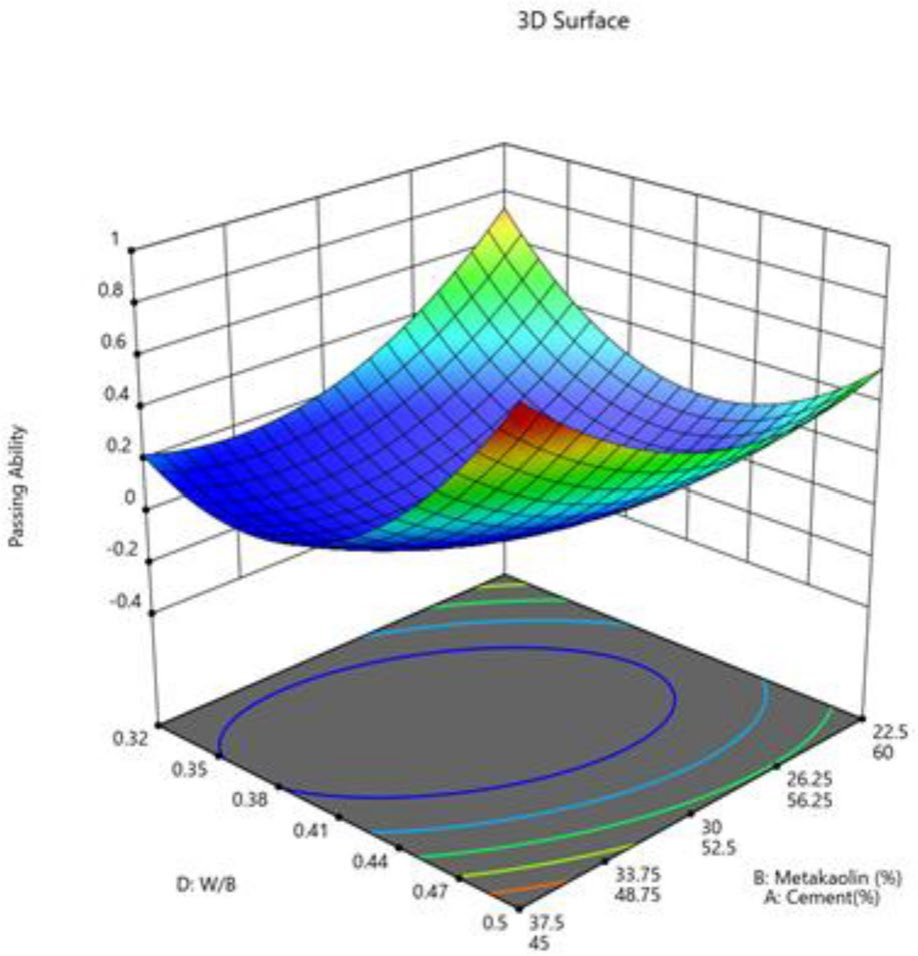
Combine effect of factors cement, metakaolin, W/B ration on passing ability.

The Model F-value of 46.57 suggests that the model is significant. Only 0.01% of the time may noise be the cause of an F-value this high. When the p-value is less 0.0500, model terms are deemed significant. If the value is higher than 0.1000, model terms are not significant. If your model has many extraneous terms (apart from those required to maintain hierarchy), model reduction may improve it. Fit statistics show that there is less than a 0.2 discrepancy between the Predicted R^2^ of 0.8515 and the Adjusted R^2^ of 0.9551. Adeq Precision measures the signal-to-noise ratio. The ideal ratio is at least 4. Your signal strength ratio of 20.475 points is adequate. The design space can be explored using this model. Figures 14, 15, 16, 17 shows the combined impact of two factors on passing ability.

Passing ability = + 1.33A + 0.2537B − 0.0808C − 0.3246D − 3.89AB + 0.9066BD + 0.4144D^2^ + 0.1093F^2^.

#### Segregation resistance

**Figure 18:**
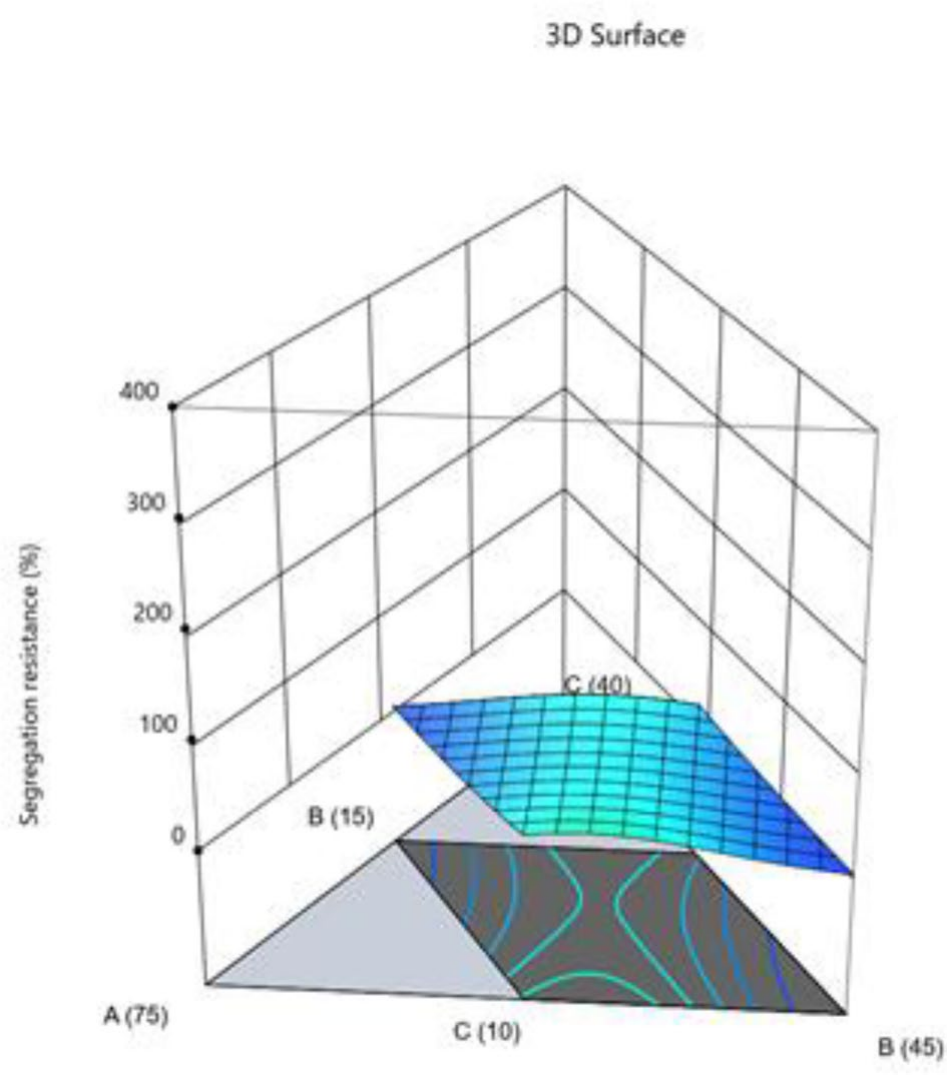
Combine effect of (**A**) cement, (**B**) metakaolin, (**C**) lime powder on segregation resistance.

**Figure 19:**
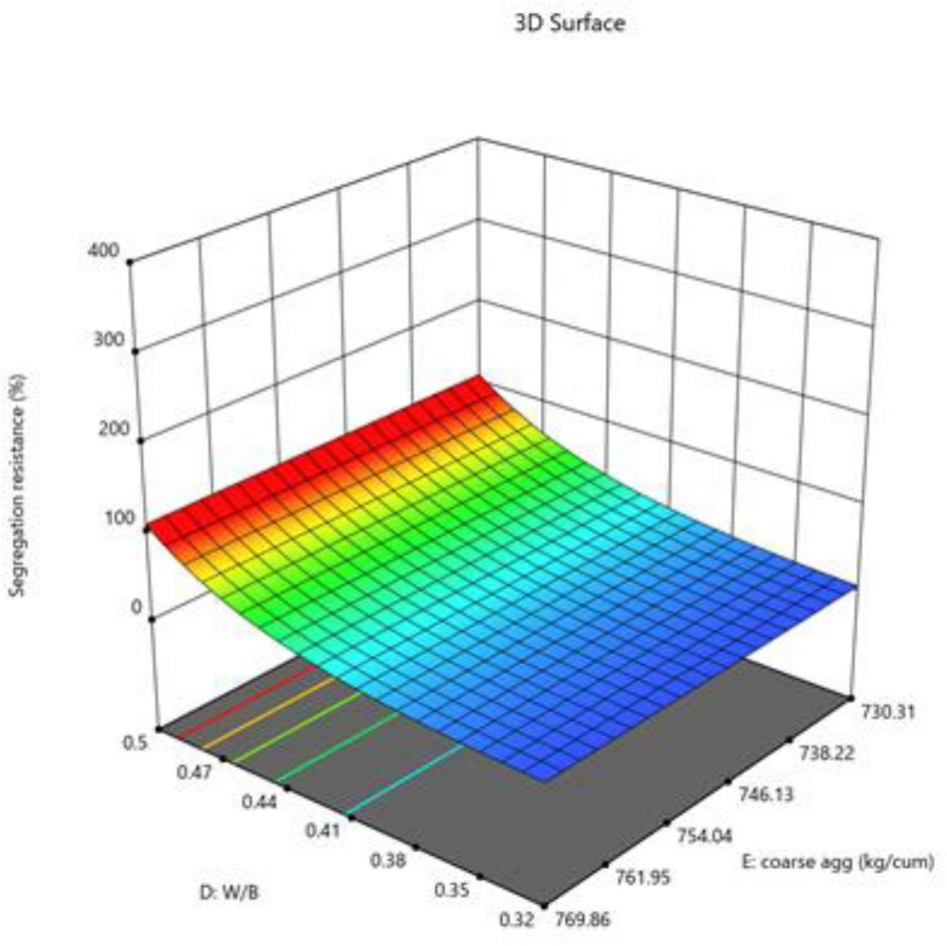
Combine effect of W/B ratio and coarse aggregate on segregation resistance.

**Figure 20:**
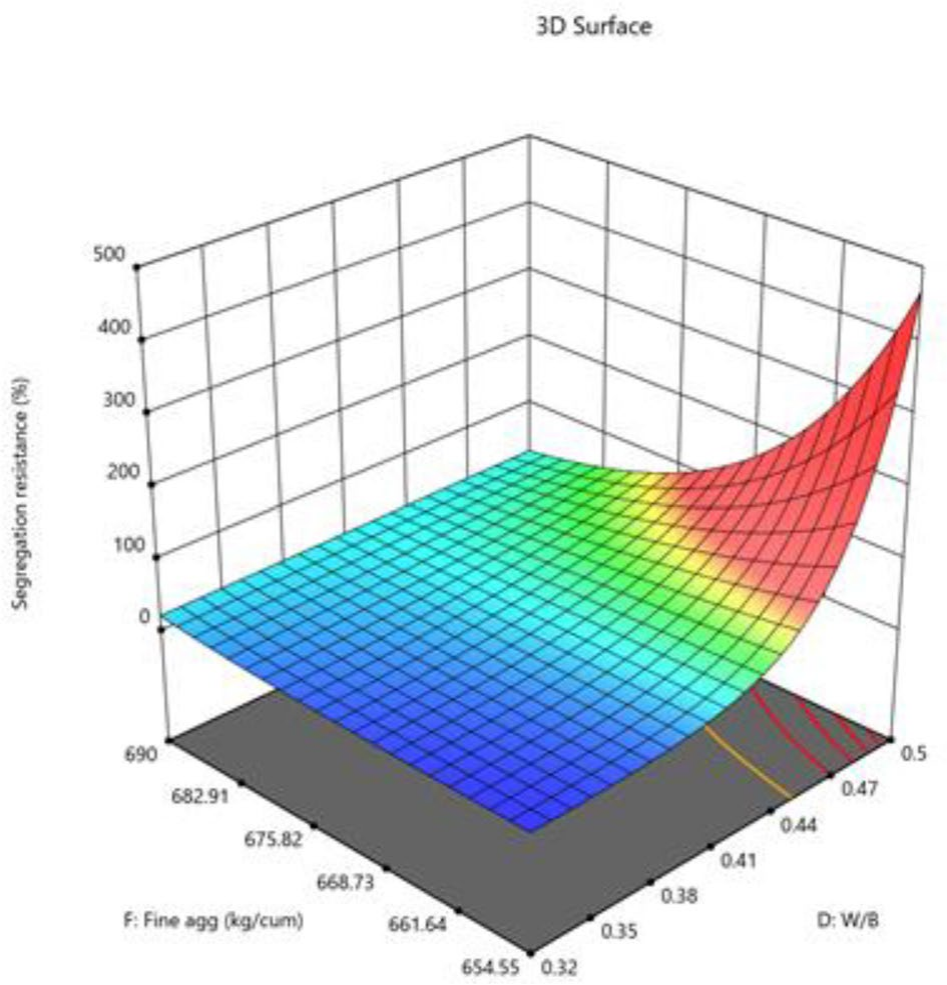
Combine effect of W/B ratio and fine aggregate on segregation resistance.

The model is implied to be significant by the model's F-value of 5.24. An F-value this large may result from noise just 1.28% of the time. Model terms are considered significant when the p-value is less than 0.0500. Model terms are not significant if the value is higher than 0.1000. Model reduction may enhance your model if it has many unnecessary terms (excluding those necessary to maintain hierarchy). The Predicted R^2^ of 0.4792 and the Adjusted R^2^ of 0.5857 are reasonably in agreement; that is, the difference is less than 0.2. The ratio of signal to noise is measured by Adeq Precision. A ratio of at least 4 is preferred. Your signal is strong enough based on your ratio of 7.893. To move around the design space, utilize this model. The segregation resistance data was converted to log base 10 before being used. The combined impact of two factors on test-taking performance is presented in Figs. 18, 19, 20.

$${\text{Log}}_{{{1}0}} ({\text{segregation}}\;{\text{ resistance}}\;(\% ) = - 0.{\text{7391A}} + 0.{365}0{\text{B}} + {1}.{\text{69C}} + 0.{\text{6778D}} + {5}.{\text{54AB}} - 0.{623}0{\text{DF}}$$

Final Equation in Terms of L-Pseudo Components and Coded Factors.

#### Compression strength at 7 days

**Figure 21:**
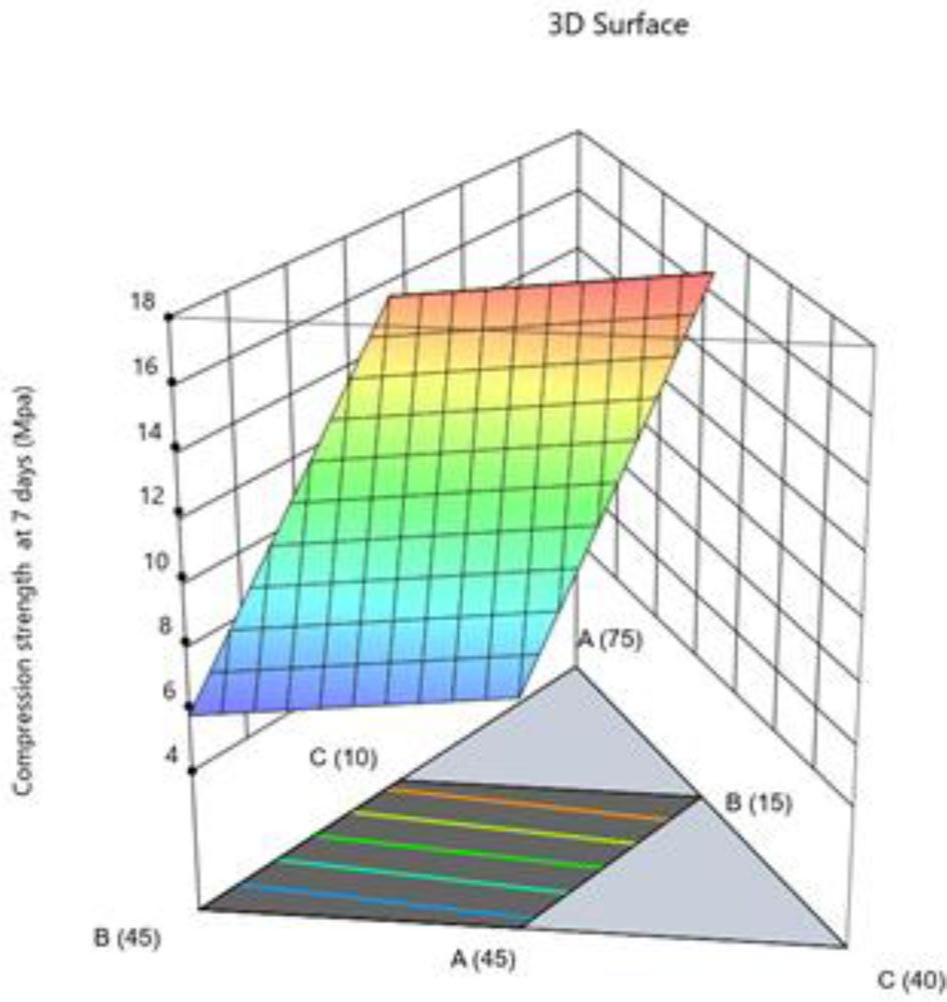
Combine effect of (**A**) cement, (**B**) metakaolin, (**C**) lime powder on compression strength 7 days.

**Figure 22:**
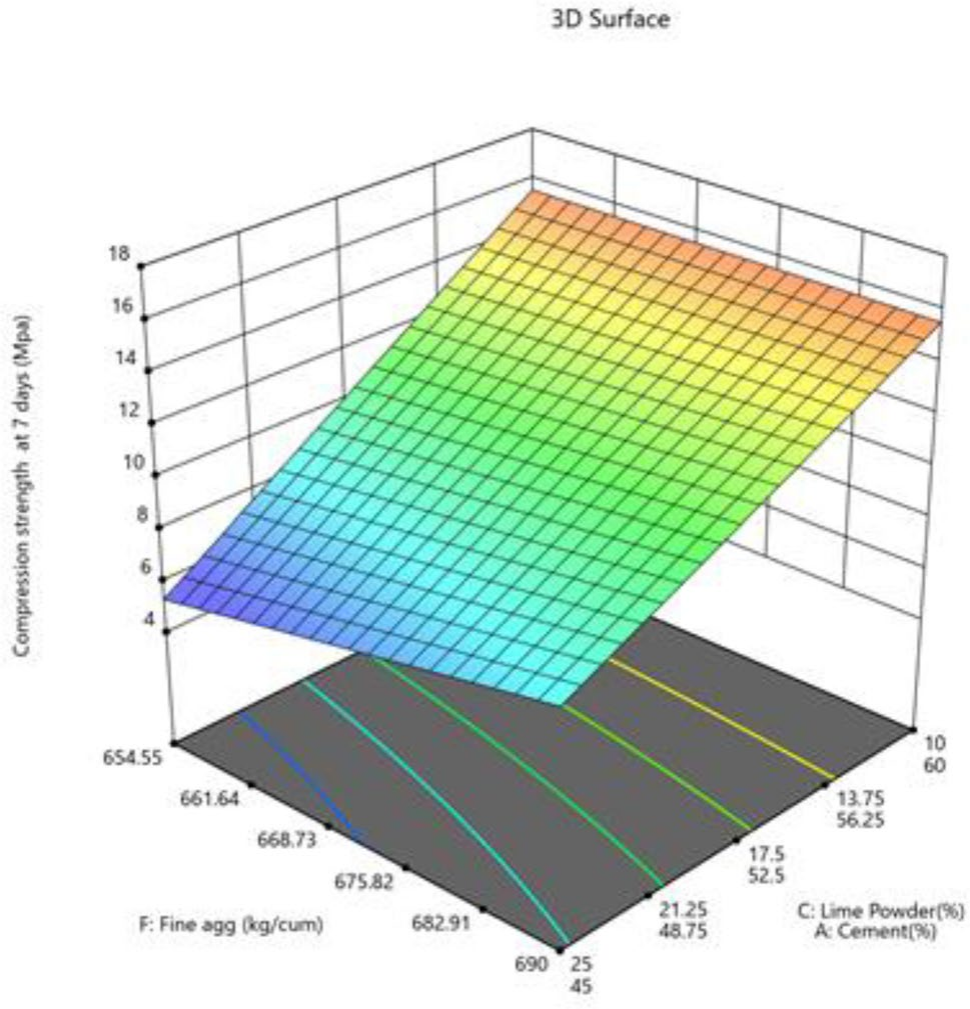
Combine effect of factors fine aggregate, cement, lime powder on compression strength 7 days.

The model is suggested to be significant by the Model F-value of 48.08. An F-value this large might happen owing to noise only 0.01% of the time. Model terms are considered significant when the p-value is less than 0.0500. Model terms are not significant if the value is higher than 0.1000. Model reduction may enhance your model if it has a large number of unnecessary terms (excluding those necessary to maintain hierarchy). According to fit statistics, the difference between the Predicted R^2^ of 0.8705 and the Adjusted R^2^ of 0.9040 is less than 0.2. The ratio of signal to noise is measured by Adeq Precision. A ratio of at least 4 is preferred. Your signal is strong enough based on your ratio of 17.542. To move around the design space, utilize this model. Impact of two parameters combined on compression strength 7 days presented in Figs. 21 and 22.

Final Equation in Terms of Pseudo Components and Coded Factors

$${\text{Compressive}}\;{\text{ strength }}\;{\text{at}}\;{ 7}\;{\text{ days}}\; \, ({\text{MPa}}) = \, + {25}.{3}0{\text{A}} + {5}.{\text{81B}} + {8}.0{\text{6C}} + {3}.{\text{24CF}}$$

#### Compression strength at 28 days

**Figure 23:**
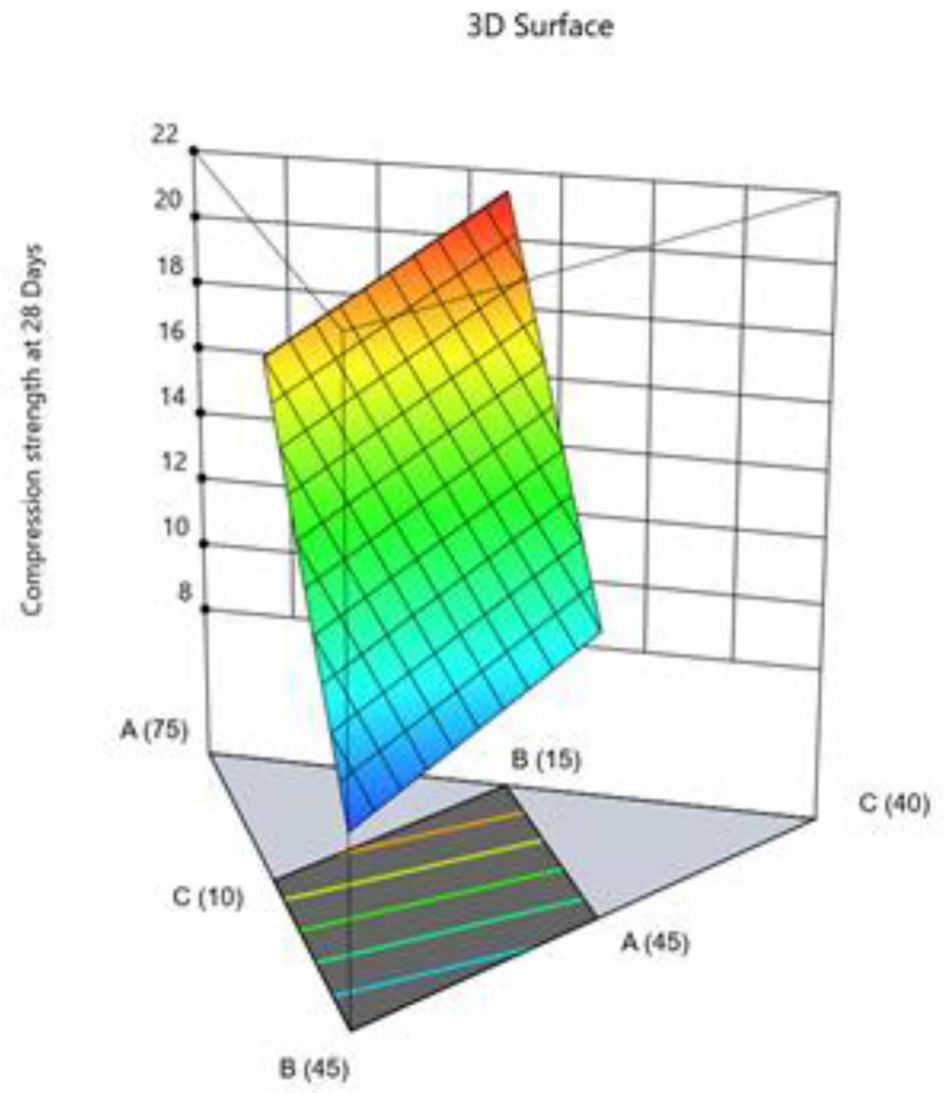
Combine effect of (**A**) cement, (**B**) metakaolin, (**C**) lime powder on compression strength 28 days.

**Figure 24:**
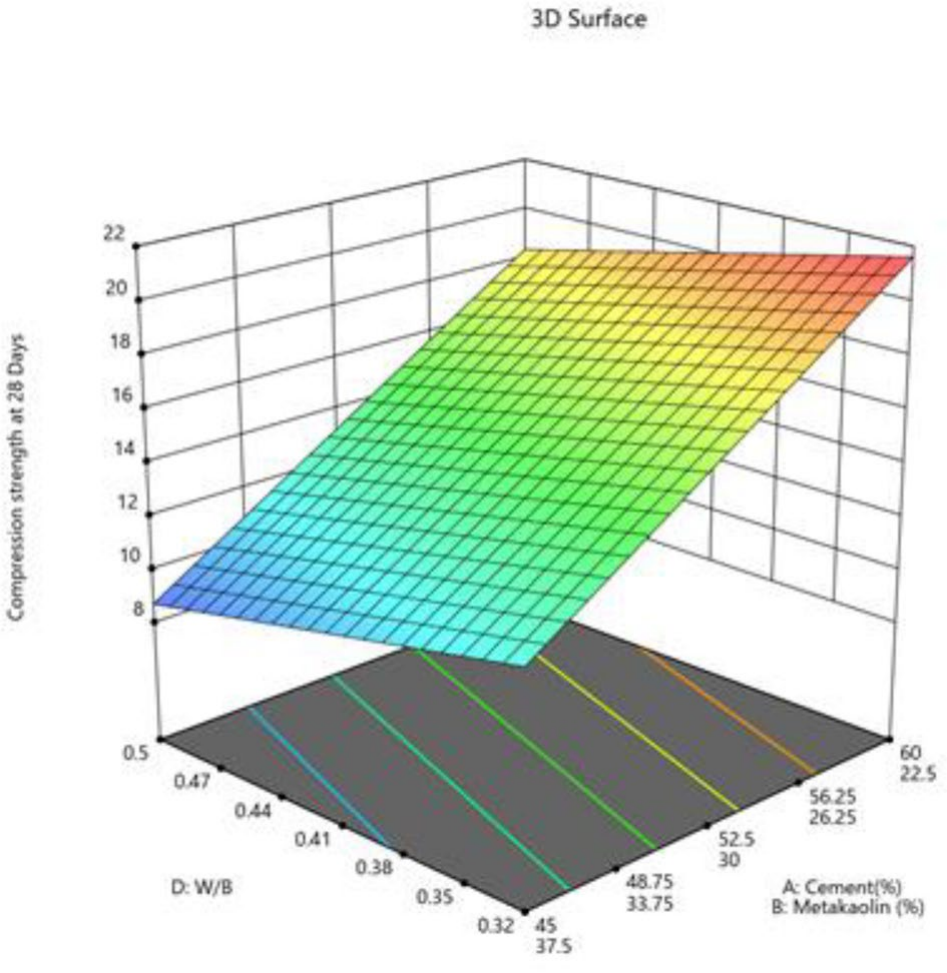
Combine effect of cement, metakaolin, W/B ratio on compression strength 28 days.

The value 30.09 Model F indicates significant model. An F-value this large might happen owing to noise only 0.01% of the time. Model terms are considered significant when the p-value is less than 0.0500. Values greater than 0.1000 showed that the model's terms were not significant. If there are an excessive amount of non-significant model terms present, then a model reduction might be beneficial. The difference is less than 0.2 between the Predicted R^2^ of 0.8065 and the Adjusted R^2^ of 0.8533. The signal to noise ratio is estimated by Adeq Precision, and a ratio greater than 4 is preferred. Your signal is strong enough based on your ratio of 13.305. To move around the design space, utilize this model. Final Equation in Terms of Coded Factors and L_Pseudo Components. The combine effect of two factors on compression strength 7 days presented in Figs. 23 and 24.

$${\text{Compressive}}\;{\text{ strength }}\;{\text{at }}\;{28}\;{\text{ days}}\;({\text{MPa}}) \, = \, + {28}.{\text{27A}} + {8}.{\text{91B}} + {14}.{\text{58C}} - {1}.{\text{62D}}$$

**Figure 25:**
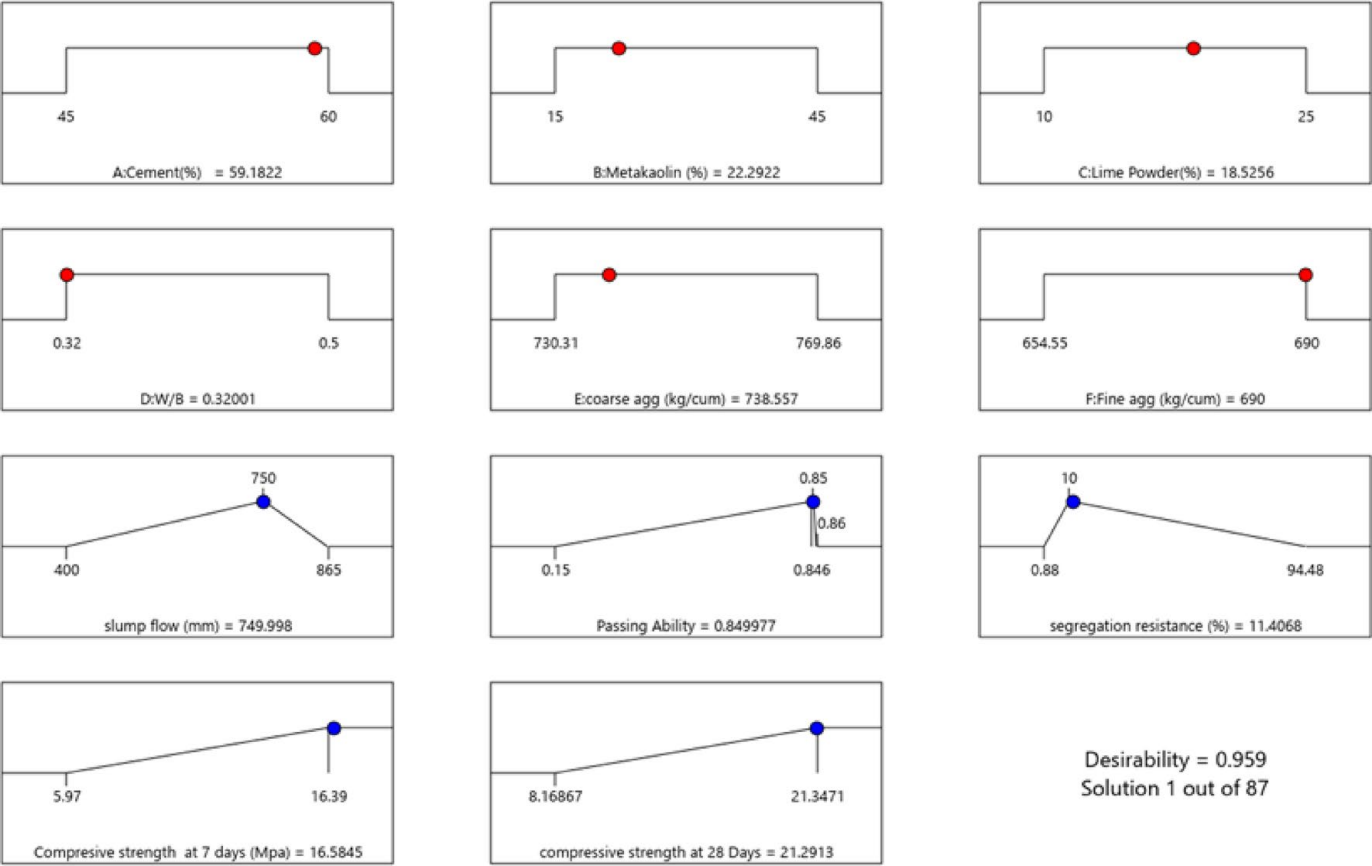
Desirability analysis.

The optimized mix design predicted by software has shown that desired features were attained at following ratio of input parameters including cement replacement level 41.22% by 22.21% metakaolin and 19.08% LP respectively keeping w/b ratio at 0.376, coarse aggregate at 736.143 kg/cum, fine aggregate at 689.99 kg/cum. The mix design was validating practically according to composition generated by software, and it was observed that predicted responses were in agreement with observed responses in terms of slump flow 750.0 mm, passing ability 0.85, segregation resistance 11.70%, compressive strength at 7 days and 28 days 16.41 MPa and 21.12 MPa respectively. The desirability was shown in Fig. 25, where ramp showing the optimized conditions for the desired properties.

## Conclusion

As the global demand for concrete continues to rise^48,49^, implementing sustainable and cost-effective solutions becomes increasingly critical. The findings of this study pave the way for wider adoption of RSM-optimized SCC mixes with high levels of SCMs. The study highlights:

Through RSM optimization trials, it was determined that an optimal mix design included at least 41.22% cement replacement, along with 22.21% metakaolin and 19.088% limestone powder, with specific aggregate ratios and 0.376 water-to-binder ratio.

The study demonstrates the validity of the predictive approach utilized, as the experimental results closely align with the outcomes predicted by the model. This indicates a satisfactory experimental design for measuring the properties of new SCC mixes, enhancing confidence in the accuracy and reliability of the results obtained.

The utilization of metakaolin and limestone powder as cement substitutes up to a maximum level of 41% is deemed cost-effective, sustainable, and eco-friendly. This approach not only contributes to reducing environmental impact but also offers economic benefits.

Findings also suggest that SCC incorporating metakaolin exhibit enhanced performance in terms of filling ability, passing ability, and resistance to segregation. Moreover, the use of metakaolin leads to improvements in both early-age and long-term compressive strength. This indicates the potential for utilizing MK-based SCC in structures requiring high early strength gain.

Additionally, the limited awareness and conventional attitudes toward adopting new materials in the construction industry pose challenges to the acceptance of research findings. However, as an initial step, this optimized mix design holds significant promise for the precast industry. The decreased reliance on costly Ordinary Portland Cement (OPC) results in substantial cost savings, thereby encouraging precast fabricators. Moreover, the substitution of OPC with supplementary cementitious materials (SCMs) such as Metakaolin and Lime powder not only reduces the environmental footprint of precast concrete production but also underscores the superior performance of SCC mixes with high SCM content. These mixes exhibit excellent filling ability, passing ability, and segregation resistance, which are critical properties for precast applications.

## Future research

Future research should investigate (i) the lifecycle environmental impacts of SCC mixes with high SCM content to ensure overall sustainability, and (ii) field trials and long-term monitoring to assess durability, and overall performance especially against the creep loading.

## Data Availability

All data used in this research appear in the submitted article.
